# Improving the composition of donor milk using machine learning and optimisation techniques

**DOI:** 10.1371/journal.pone.0345653

**Published:** 2026-03-24

**Authors:** Jacqueline Muts, Danée Knevel, Dick den Hertog, Rachel K. Wong, Timothy C.Y. Chan, Britt J. van Keulen, Johannes B. van Goudoever, Chris H.P. van den Akker

**Affiliations:** 1 Department of Pediatrics, Emma Children’s Hospital. Amsterdam UMC, University of Amsterdam, Amsterdam, The Netherlands; 2 Amsterdam Reproduction & Development research institute, Amsterdam UMC, Amsterdam, The Netherlands; 3 Department of Business Analytics, Faculty of Economics and Business, University of Amsterdam, Amsterdam, The Netherlands; 4 Department of Mechanical and Industrial Engineering, University of Toronto, Toronto, Ontario, Canada; 5 Department of Neonatology, Emma Children’s Hospital. Amsterdam UMC, University of Amsterdam. Amsterdam, The Netherlands; North-Caucasus Federal University - Pyatigorsk Campus: Severo-Kavkazskij federal'nyj universitet Patigorskij institut filial, RUSSIAN FEDERATION

## Abstract

**Background and aims:**

The macronutrient composition of donor human milk (DHM) can vary substantially due to several factors such as maternal age, diet, and lactation duration. However, consistent macronutrient levels in DHM facilitate the administration of the required amounts to preterm infants. The current pooling strategy at most human milk banks combines milk from different batches from a single donor. This study aims to stabilize the macronutrient quality of DHM by pooling milk from different donors by utilizing machine learning prediction and optimisation techniques.

**Methods:**

The current pooling strategy is compared with a new theoretical approach that pools milk batches from up to 5 donors. To predict the crude protein and energy content, we used the following variables: body mass index, the donor’s diet (vegetarian or non-vegetarian), maternal age, full-term or preterm delivery, lactation stage, and volume pumped. These predictions are then used within an optimisation model to create milk pools that minimize the deviations from the target macronutrient levels (1.0 g protein/100 mL and 70 kcal/100 mL).

**Results:**

The prediction model is based on 2236 created single-donor pools from 480 donors. Random forest regression models provided the most accurate predictions of macronutrient content. The new pooling strategy using multiple donors shows reduced deviations from target values compared to the current single-donor approach (average total absolute deviation 0.402 versus 0.664).

**Conclusion:**

This study proves the potential of data-driven methods to improve operational efficiency in human milk banks, and improving the consistency of donor human milk.

## Introduction

Human milk plays a crucial role in the care of preterm infants, offering vital nutrients essential for their development [1,2], and reducing the risk of severe infections like necrotizing enterocolitis as compared to preterm formula milk [3]. In cases where mother’s own milk (MOM) is insufficiently available, donor human milk (DHM) is recommended as the first alternative [4,5]. The macronutrient composition of DHM varies substantially due to factors such as maternal age, diet, and the duration of lactation [6,7]. For instance, protein concentrations are known to decrease over time [1], while maternal diet may influence the fatty acid composition and overall macronutrient content of human milk [8,9]. Additionally, studies have highlighted individual variability among mothers and the impact of both the postpartum lactation period and gestational age on nutrient levels [10]. These findings underscore the important influence of various maternal factors on the macronutrient content of human milk.

Currently, the vast majority of DHM banks in Europe (80%) prepare bottles with milk from a single mother, as this is recommended for safety reasons, including infection prevention, biovigilance, and traceability [11,12]. However, preterm infants may receive DHM from multiple consecutive donors during their admission to a neonatal intensive care unit [12]. This may result in varying and changing nutrient delivery with subsequent effects on growth and development. It has therefore been suggested that strategies like pooling milk from multiple donors in a DHM bank could help ensure a more stable end-product, facilitating NICU’s to provide a more constant nutritional support for infants [10,13,14]. Research has shown that pooling strategies aimed at balancing nutrient levels can improve the macronutrient content and bioactive components of donor milk as well [14,15]. While milk pooling can be done randomly, it can also be done more targeted, based on actual measurements of unpooled milk or based on baseline characteristics.

Hitherto, few studies have shown the potential of machine learning and optimisation techniques for improving milk pooling strategies in DHM banks. For instance, Chan et al. employed machine learning models to predict fat and protein content based on retrospectively available covariates and then used optimisation models to determine donations to pool together to improve adherence to macronutrient targets [16]. Similarly, Wong et al. developed models to forecast macronutrient content using various donor and donation-specific variables [17]. Additionally, Sun et al. applied optimisation for targeted pooling techniques when macronutrients were known beforehand [18]. These studies demonstrate the feasibility and effectiveness of using data driven approaches in a human milk bank setting.

The aim of this study is to adapt both the prediction and optimisation approaches in Chan et al. and Wong et al. for the Dutch National Human Milk Bank, and to analyse whether the resulting approach leads to improvements for the Dutch National Human Milk Bank. Results of this study could help in achieving more stable macronutrient levels in DHM.

## Methods

This study was conducted in the Dutch National Human Milk Bank, housed in the Amsterdam University Medical Center, The Netherlands. This DHM bank currently serves all neonatal intensive care units in The Netherlands, offering DHM for very preterm infants (gestational age < 30 weeks) whose mothers cannot produce sufficient milk. We utilized an existing database, accessed on 3rd April 2024, which contained information on all new donors accepted to donate to our DHM bank between March 2017 and April 2024. Data was collected regarding each donor’s postpartum donation timeline, duration, and expressed amounts. Further data collected included the donor’s age, BMI, and dietary information. In addition, information is collected about the infant, including its gestational age.

The research we conducted only utilizes routinely collected data which were provided by the healthy donors who registered voluntarily at our DHM bank. All of these data are required for the day-to-day operation of the donor milk bank. No additional data has been collected nor have any further analyses been performed on the milk for this study specifically. The data used in this research was pseudonymized to protect donor identities. As such, our study does not fall under the scope of the Dutch Act on Medical Research Involving Human Subjects. Donors are, however, asked to sign a waiver statement at each milk donation, which includes an optional consent question whether their milk may be used in scientific research. We only included results from volunteers who gave permission to do so.

All potential milk bank donors undergo an approval process to ensure that they meet the stringent requirements for donation. When donating, women are instructed to express the total milk content from one of both breast(s) instead of expressing the fore- or hindmilk solely. Donated milk batches are stored at −20°C for no more than 3 months before further processing.

The current practice of our DHM bank is to combine individually expressed milk donations (range ~50–250 mL), from the same donor pumped on different adjacent dates, into larger 2-litre pools. The DHM bank uses a regularly calibrated mid-infrared human milk analyzer (HMA, MIRIS®, Uppsala, Sweden) to measure protein, fat and carbohydrate content in a sample from each 2-litre pool as a quality measure. The HMA measures true protein by assessing total nitrogen, then adds approximately 20% to account for non-protein nitrogen, resulting in an estimate of crude (total) protein content. This study only reports the crude protein concentration rather than the true protein content. Energy content is calculated by applying Atwater factors to the measured concentrations of these macronutrients.

### Objective

Our objective was to develop a model that could advise us which frozen milk bottles available in the freezers should be combined into a 2-litre pool, in order to achieve the most homogenous protein and energy content. Therefore, a machine learning model was trained with a dataset of potentially confounding variables as listed above. Although the study was retrospective in nature, a real-life situation was mimicked in that the model could only choose from available bottles that had truly arrived in our freezers at the time of creating a 2-litre pool.

### Data analysis

To better understand the factors affecting the protein and energy content of human milk, we examined various donor characteristics and their correlations with milk composition. As previously mentioned, factors that can influence the macronutrient content of human milk include the donor’s BMI at their first donation, lactation period (measured in days postpartum), average volume of milk per expression, gestational age, maternal age, and maternal diet (vegan/vegetarian or omnivorous). To explore the relationship between these donor characteristics and the protein and energy content of the donated milk, multiple linear regression analyses and the Pearson correlation test were performed. Statistical significance for the multiple linear regression and Pearson correlation analyses was defined as a p-value less than 0.05. No correction for multiple testing was applied, as the analyses were exploratory in nature and aimed at identifying potential patterns rather than testing predefined hypotheses.

### Prediction model

The study utilized linear regression [19], Lasso LARS [20], random forest [21], gradient boosting [22–24], AdaBoosting [25], SVM [26], and Ensemble [24] models to capture complex data relationships and improve predictive performance. Data for protein and energy content were split into training (80%) and testing (20%) sets, with GridSearchCV employed to fine-tune model hyperparameters for optimal performance, ensuring precise scaling and regularization where necessary [27]. More details on these machine learning models can be found in the S1 Text (Section 1).

To evaluate the performance of each prediction model, the mean absolute error (MAE), root mean square error (RMSE), and mean absolute percentage error (MAPE) were calculated using the test set predictions. For each model, the MAE was computed as the mean of the absolute differences between the actual and predicted values. The RMSE was determined as the square root of the average of the squared differences between the actual and predicted values. The MAPE was calculated as the mean of the absolute percentage errors, multiplied by 100 to express the error as a percentage of the actual values. Additionally, the standard deviation of the cross-validated test scores was extracted from the GridSearchCV results to assess variability in the model’s performance. This step was crucial to understanding the consistency of the model’s predictions across different training data subsets.

### Optimisation model

The mathematical model for optimising the pooling process at the DHM bank relies on three key sets: the actual available bottles in the freezer at the moment of pooling, the set of donors corresponding to the available bottles, and lastly the set of pools that have to be made. We determined that the protein and energy content should be stabilised around the target values of 1.0 g/100 mL and 70 kcal/100 mL, respectively. These targets were established by analysing the average macronutrient composition of all available samples in our database. In defining these values, we also considered the practical feasibility of producing a standardized product.

The requirements that the model had to follow were based on the pooling guidelines of the Dutch National Human Milk bank. For example, each pool should be close to or equal to 2 liters, which is the capacity of the pooling bottles. To operationalize this, the model ensures that each pool contains at least 2.00 liters and no more than 2.05 liters. This upper limit allows for slight variations in bottle capacity and provides a small margin of flexibility while still adhering to the intended specifications. Additionally, each pool should contain milk from three to five different donors. To ensure no bottle is used in more than one pool, constraints were included to guarantee each bottle is assigned to only one pool. From a practical perspective, bottles cannot be split without thawing, which would risk contamination and reduce milk quality. Therefore, assigning each bottle to a single pool improves traceability and maintains safety standards essential for human milk banking [11]. Furthermore, the model prioritizes using bottles nearing their expiration dates, thereby minimizing waste by ensuring these bottles are used before they expire (stored for no more than 3 months [28]). To manage these requirements, we employed decision variables to ensure the proper selection of bottles of milk from various donors. The primary decision variable indicates which bottle was chosen for a pool. Another binary variable indicates whether a donor was used in a pool. For a detailed description of the optimisation model, we refer to the S1 Text (Section 2).

For sensitivity analyses the model was tested with different numbers of pools (ranging from 2 to 15) to evaluate how this factor impact optimisation results. The objective value reflects the total deviation from the target crude protein and energy contents across all pools.

## Results

Between March 2017 and March 2024, the DHM bank received milk from 480 new donors and from their donations 2236 2-liter single-donor pools of milk were created.

### Factors influencing protein content

Donor BMI, the number of days postpartum and donor age all showed a negative correlation with the protein content (all p < 0.001) (Table 1 & S1 Fig in S1 File). No statistically significant correlation was found with the Pearson correlation test between the average volume of milk expressed by the donor that was in a 2-liter pool and its protein content (Table 1).

**Table 1:** Pearson Correlations (p-values) between protein, energy, and other covariates.

	Crude protein (g/100 mL)	Energy (kcal/100 mL)
Crude protein (g/100 mL)	–	0.16 (<0.001)
Energy (kcal/100 mL)	0.16 (<0.001)	–
Days postpartum	−0.20 (<0.001)	0.17 (<0.001)
Volume pumped (mL)	0.01 (0.559)	−0.15 (<0.001)
BMI (kg/m^2^)	−0.10 (<0.001)	0.05 (0.013)
Premature (yes/no)	0.06 (0.003)	−0.01 (0.612)
Donor age (y)	−0.10 (<0.001)	0.17 (<0.001)
Vegetarian or Vegan donor (yes/no)	0.05 (0.020)	−0.01 (0.514)

Values are presented as Pearson correlation coefficients with the corresponding p-values in parentheses. The Pearson correlation coefficient indicates the strength and direction of the relationship between variables, where ‘0’ represents no correlation, ‘1’ indicates a perfect positive correlation, and ‘-1’ indicated a perfect negative correlation.

### Factors influencing energy content

Maternal BMI and age, as well as the days postpartum, all showed a statistically significant positive correlation (p = 0.013 and p < 0.001 and p < 0.001, respectively) with the energy content (Table 1 & Fig S2 in S1 File). Furthermore, a statistically significant negative correlation was found between the volume of milk pumped and the energy content (p < 0.001) (Table 1 & Fig S2 in S1 File).

### Term versus preterm milk

The dataset contained 1,670 milk pools formed from donors whose infants were born full term, and 566 instances in which the milk was from donors with infants born preterm, i.e., before 37 weeks of gestation. A statistically significant correlation was found between gestational age (coded as 0 = full-term and 1 = preterm) and the protein content of milk (r = 0.06, p = 0.003), suggesting that the protein content tends to be higher in milk form donors with preterm infants. However, no difference was observed in the energy content of the milk (Table 1 & Fig S3 in S1 File).

### Diet

From our cohort, 269 milk pools were obtained from donors adhering to a vegetarian or vegan dietary pattern. A statistically significant correlation was found between diet type (coded as 0 = omnivorous, 1 = vegetarian) and the protein content of milk (r = 0.05, p = 0.020), suggesting that protein content tends to be higher in milk from vegetarian donors. No statistically significant difference was found between the dietary pattern and the energy content (Table 1 & Fig S4 in S1 File).

### Protein and energy content prediction

All variables included in this study contributed to the predictive performance and were therefore retained in the final model: days postpartum, average daily pumped volume, donor BMI, gestational age category (preterm or term birth), donor age, and diet (vegetarian or omnivorous).

The random forest regression model achieved the lowest MAE of 0.112 with a standard deviation of 0.040 for predicting the protein content, indicating not only high accuracy but also consistent performance across different data subsets. The relatively low standard deviation suggests that the model’s predictions remain stable regardless of the specific subset of data used for training and validation. This model also had the lowest RMSE value at 0.155, indicating the highest accuracy (Table S1 in S2 File).

**Fig 1:**
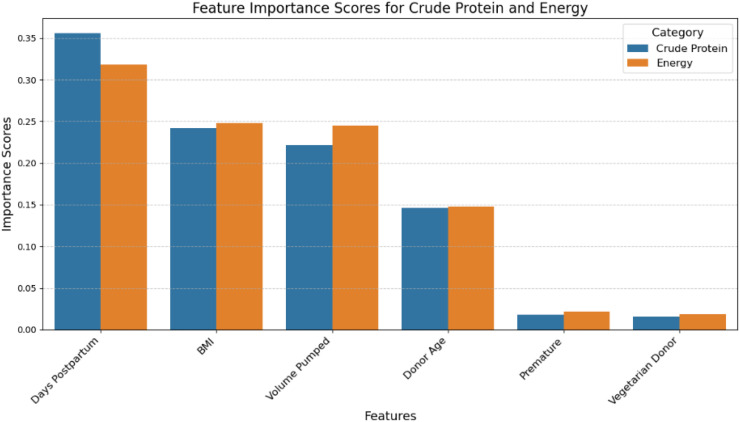
Feature importance scores from Random Forest models for predicting Crude Protein and Energy content.

With regard to the prediction of energy content in DM pools, the random forest regression model also achieved the lowest MAE of 5.34 with a standard deviation of 0.038 and an RMSE of 7.231, indicating that this model again performs better in predicting the energy content compared to other models (Table S2 in S2 File). Comparing the model outcomes for predicting both protein and energy revealed that the random forest regression models consistently outperformed all other models in both instances. As a result, we chose the random forest regression as the final model, and we used its key metrics to assess which features were most important for the predictions (Fig 1).

### Optimisation model

The objective of the optimisation model was to minimise deviations in the nutritional content of the pooled milk from the target values for crude protein and energy. The optimal solution achieved an average objective value of 0.402, representing the average minimised total absolute deviation across all pooling scenarios. This small deviation indicates that the pooled milk closely matched the target values of 1 g/100 mL for crude protein and 70 kcal/100 mL for energy, ensuring that the final product was of consistent nutritional quality. More details on the solver used, the time needed to solve the model, and the accuracy of the final solution can be found in S1 Text (Section 3).

### Comparison to current practice

Compared to the current practice of using milk from a single donor per pool, the optimisation model, using between three and five donors, showed an improvement in the nutritional consistency of the pooled donor milk. The current practice resulted in an average total absolute deviation of 0.664, considerably higher than the optimised model’s 0.402. In other words, the optimisation approach resulted in a 39% improvement in stability, reducing the deviation and thereby enhancing the overall performance. This means that pooling from multiple donors allowed for better control over the consistency in nutritional content of the pooled milk. However, when milk from multiple donors is pooled, the shelf life of the pool is determined by the bottle with the shortest remaining time before expiration. As a result, the pooled milk must be used before the earliest expiration date among its components, which can lead to a shorter remaining shelf life compared to using milk from a single donor (S5 Fig in S1 File). While the current practice did occasionally achieve results close to the target values by chance, these were less consistent than those from the optimized pooling strategy. This suggests that expanding the number of donors per pool can help stabilize the nutritional content of donor milk.

### Robustness analysis

The robustness analysis evaluated the stability and performance of the optimisation model under worst-case scenarios. This analysis is particularly important for the crude protein and energy content values of individual bottles, as they are predictions and therefore uncertain parameters. In this worst-case scenario, the optimisation model aimed to maximize the deviations from the target values. The deviations were much larger than in the initial optimisation, with an average objective value of 1.113 (S6 Fig in S1 File). Despite this, the model was still able to generate pools with nutritional values that remained within the predefined MAE bounds used in the robustness analysis, even though these values often fell below the target levels. These results demonstrate that the model is robust, as it consistently produced feasible and optimal solutions under worst-case uncertainty in input parameters, while preserving acceptable predictive accuracy.

### Sensitivity analysis

To evaluate how variation in pool number affects the optimisation results, the model was run with varying numbers of pools, from 2 to 15 pools. The normalised objective, the average deviation per pool, vary slightly with the number of pools but remain relatively stable with an objective between 0.03 and 0.05 per pool. This stability suggests that the model performs effectively regardless of the number of pools (Fig S7 in S1 File).

Similarly, the optimisation results were analysed for different combinations of minimum and maximum allowed donors, while keeping the number of pools constant. Overall, the objective value remained relatively stable across scenarios, with only a slight increase when fewer donors were permitted. This indicates that the model can still perform well under tighter donor constraints, but that increased donor flexibility allows for improved stabilisation of macronutrient content. In addition, it was observed that the model used a higher average number of donors per pool when the upper donor limit increased, supporting the notion that broader donor selection improves the ability to match nutritional targets (Table S3 in S2 File).

## Discussion

In this study, we aimed to develop strategies to enhance the nutritional quality and stability of DHM through the application of machine learning and optimisation techniques. By predicting the macronutrient content of human milk, we were able to optimise donor milk pools, ensuring consistent levels of protein and energy. After evaluating the performance of different prediction models, we found that the random forest regression models had the highest prediction accuracies, with the lowest MAE and RMSE values compared to other models.

The results of our study indicate that protein and energy content can be predicted with high accuracy using machine learning techniques. Specifically, our model achieved a MAE of 0.112 g/dL for protein, which falls within acceptable ranges for practical applications. These results are comparable to those reported by Wong et al. [17], who achieved an MAE of 0.1 g/dL for protein predictions in pooled donor milk samples using machine learning models. Both values are consistent with the reported measurement error associated with the MIRIS® Human MILK analyzer of 0.10 g/dL [29]. While direct comparisons are challenging due to differences in study design and datasets, our findings suggest that machine learning models can reliably predict macronutrient content in donor milk.

Subsequently, utilising a predict-then-optimise approach, the pools can be optimised based on these predictions. The results show that the pooling process can be modelled and optimised, resulting in usable and stable pools for the DHM bank. Our results compare the current strategy of using only one donor per pool against a new strategy in which multiple donors are used for one pool. In addition, stability of the pools was higher, with reduced deviations from the target values, when using multiple donors in the pools compared to the DHM bank’s current practice of using one donor per pool. Additionally, these pools contained more bottles close to their expiration date, which can improve the management of milk inventory. These findings highlight the potential for advanced data-driven methods to enhance the nutritional consistency of donor milk provided to preterm infants.

Although the standard practice in most DHM banks is to pool milk from a single donor because of simplicity and due to presumed safety concerns such as infection control, biovigilance, and traceability, our approach remains aligned with safety guidelines. In our model, milk is pooled from multiple donors only prior to pasteurisation, meaning the final product still undergoes a full Holder pasteurisation process, which is effective in eliminating potential pathogens [30]. Furthermore, appropriate documentation and pooling logs can maintain traceability even in multi-donor pools. As such, pooling milk from multiple donors with matched nutritional profiles, followed by pasteurisation, can be considered a safe and feasible alternative to single-donor pooling, with the added benefit of improved nutritional consistency.

The most significant predictor for both protein and energy in the random forest models was the number of days postpartum on which the breast milk was pumped. Other important predictors included the donor’s BMI and the average volume pumped per pool, which are supported by the literature [31]. Previous studies investigating factors influencing macronutrient content in human milk also found a correlation between mothers BMI [32,33], lactation stage [34] and milk volume [35]. Less influential features were maternal age, whether the milk was from a premature birth, and the donor’s vegetarian status. However, every feature contributed to the predictions, which highlights the importance of lactation period and individual donor characteristics in the macronutrient content of human milk.

While donor vegetarian status was a less influential factor in predicting crude protein and energy content in the model, we unexpectedly observed that milk from vegetarian donors had a higher crude protein concentration compared to milk from omnivorous donors, even though the existing literature reports no significant differences in macronutrient composition between vegetarian and non-vegetarian diets [36]. This finding was based on 272 milk samples from vegetarian donors and 1,979 samples from omnivorous donors, highlighting the relatively small sample size of the vegetarian group. One possible explanation for this difference is the limited dietary information available. Dietary classification in this study was restricted to broad categories (vegetarian, vegan and omnivorous) without detailed insight into specific nutrient intake or dietary quality. Furthermore, most of their milk samples were collected during the earlier stages of lactation, with relatively few samples representing the later stages. Since protein concentrations in human milk are known to decline over the course of lactation [31], this sampling bias may partially explain the observed higher protein content.

Comparing the robustness analysis results for the general optimisation problem highlights the model’s effectiveness and reliability. While the general optimisation problem achieved minimal deviations from the target values, the worst-case scenario presented an increase in the objective value, reflecting the model’s performance under extreme conditions. Despite the increased deviation, the model still maintained acceptable performance levels, demonstrating its robustness and suitability for practical applications. Furthermore, sensitivity analysis revealed that the model remained stable regardless of the number of pools created, consistently producing stable pools. This suggests that the model could contribute to improving operational efficiency while helping to maintain the nutritional quality of donor milk.

The results of this study align with previous research focused on the prediction and optimisation of macronutrient predictions in human milk for optimising infant nutrition. This study draws heavily on recent work by Chan et al. [16], who utilised machine learning models to predict the fat and protein content of milk deposits and to optimise the pooling process at milk banks. Our findings align with the theory by Chan et al. in that the random forest regression model would be the best predictor. However, their trial at the Rogers Hixon Ontario Human Milk Bank showed more robust results because it was conducted in a real-life scenario, where machine learning predictions and pooling strategies were directly integrated into daily milk bank procedures. Wong et al. [17] only focused on forecasting the fat and protein content of donor human milk. Their study, like ours, highlights the importance of various donor and donation-specific characteristics and provides simpler baseline prediction models for DHM banks lacking access to macronutrient analysers. Sun et al. [18] concentrated on optimising the pooling process for milk banks with macronutrient analysers, aiming to minimise operational costs while meeting production requirements. Their models did not examine uncertainty since they focused on a problem in which the macronutrients were known before pooling.

### Limitations

Several limitations should be acknowledged in this study. Firstly, a challenging task for this research was that there is currently only data available on the macronutrient content of pools of multiple donations of human milk from the same donor, and not individual bottles. Therefore, the predictions for the macronutrient content of individual bottles are made by a random forest model trained on data for pools of human milk. This required averaging features such as days postpartum and milk volume pumped, which may have introduced some inaccuracies. However, the days postpartum of the pooled samples used in this study differed by no more than a week.

Secondly, the optimisation model’s performance has not been tested in real-life scenarios in which the resulting pools are created and measured for their macronutrient content. Therefore, its practical application still needs to be validated through a real-life scenario study to confirm the methods.

Future research could explore the integration of other relevant factors that may influence milk quality, such as detailed dietary information from donors, additional macronutrients like carbohydrates and fatty acids, micronutrient composition, and genetic factors. Investigating the real-life application of the optimisation model by creating and measuring actual milk pools would provide valuable validation. Moreover, expanding the dataset to include individual bottle-level data could enhance the model’s accuracy and reliability.

## Conclusion

In conclusion, the application of machine learning and optimisation techniques has proven to be a promising approach to improving the quality and consistency of donor human milk at the Dutch National Human Milk Bank.

## Supporting information

S1 File Supplmentary Figures. (DOCX)

S2 File Supplementary Tables. (DOCX)

S1 Text Optimizing pooling process. (DOCX)
