# Supplementary material for: Improving the composition of donor milk using machine learning and optimisation techniques
Source: PLoS One. 2026 Mar 24;21(3):e0345653. doi: 10.1371/journal.pone.0345653 (PMC13012482; doi:10.1371/journal.pone.0345653)
Supplement: S1 File — (DOCX) [file pone.0345653.s001.docx]

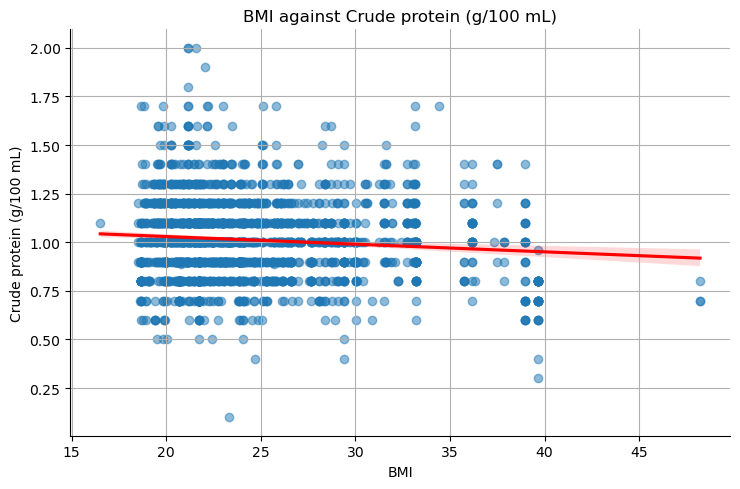

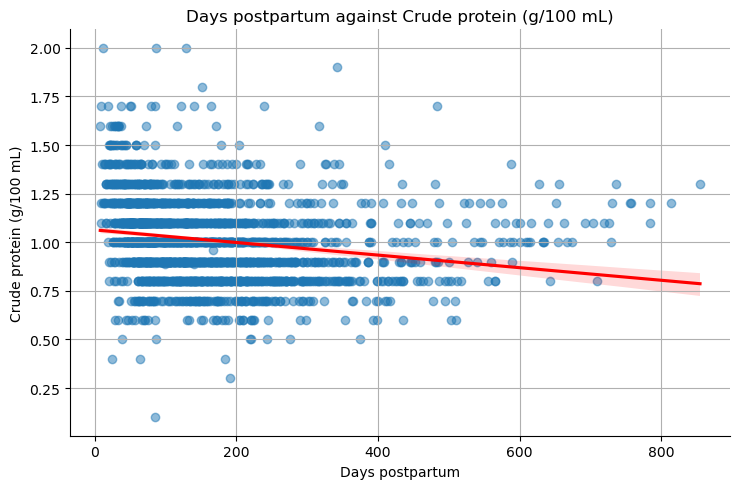


A B
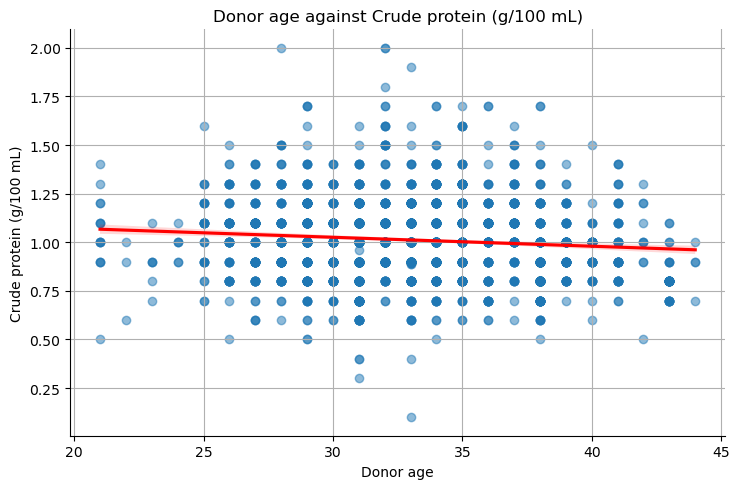

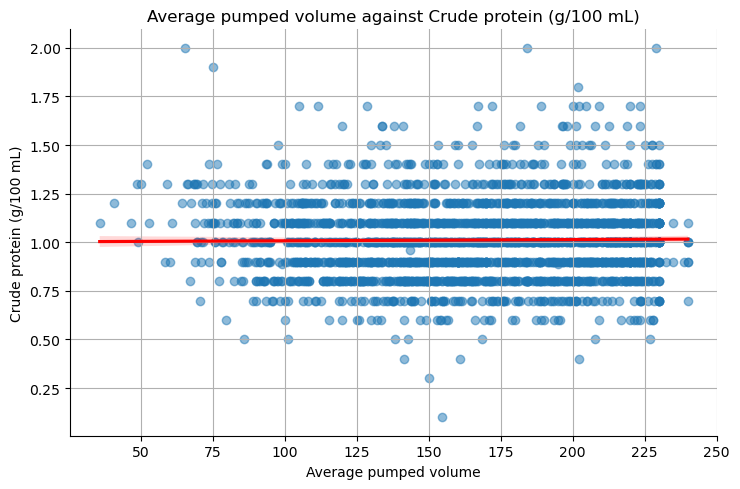


C D

**Figure 1 A-D**. Covariates against crude protein (g/100 mL). A, BMI; B, days postpartum; C, maternal age; D, average milk volume. The red line represents the correlation gradient.


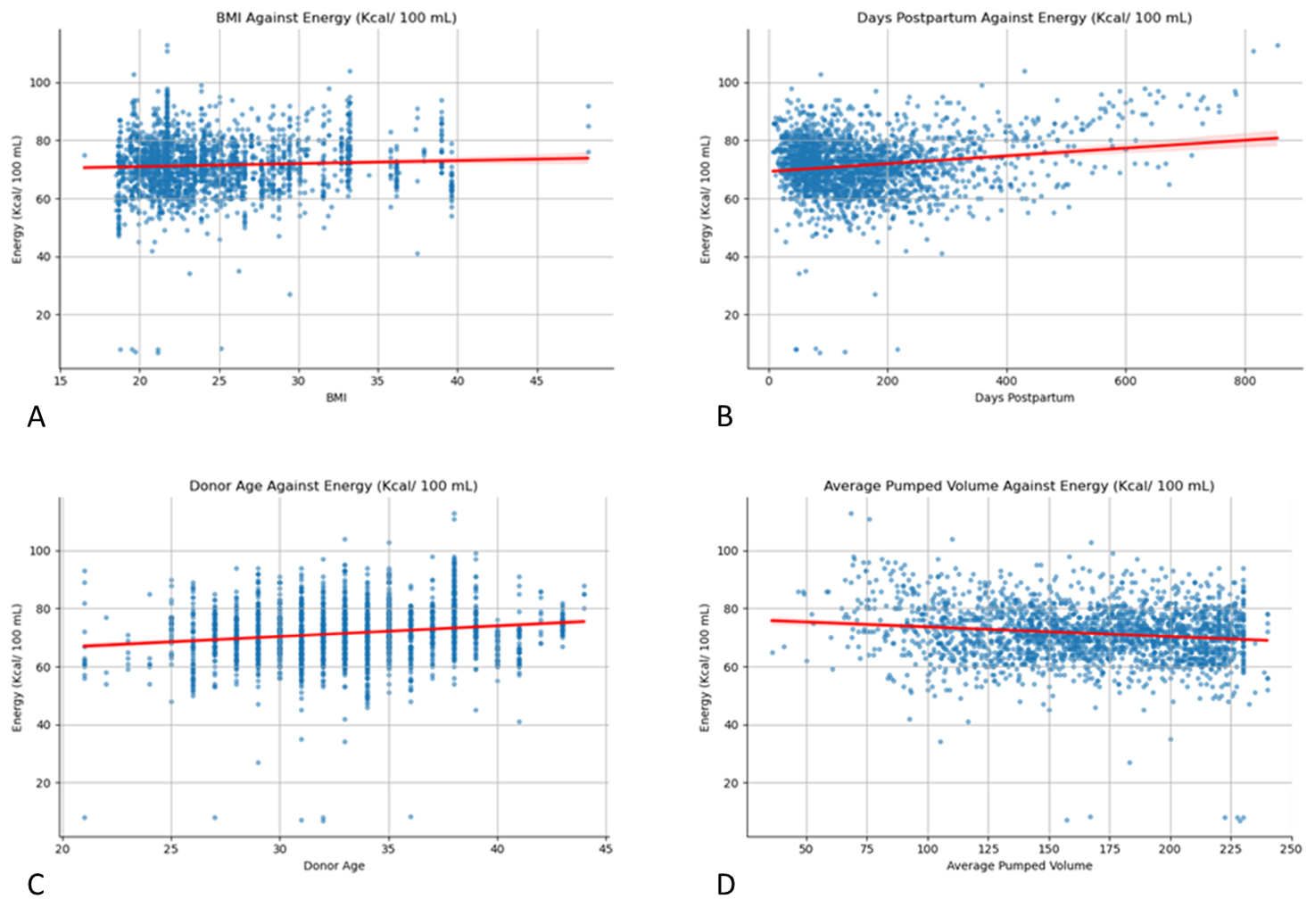


**Figure 2 A-D**. Covariates against energy (kcal/100 mL). A, BMI; B, days postpartum; C, maternal age (y); D, average milk volume per single donation (mL). The red line represents the correlation gradient.


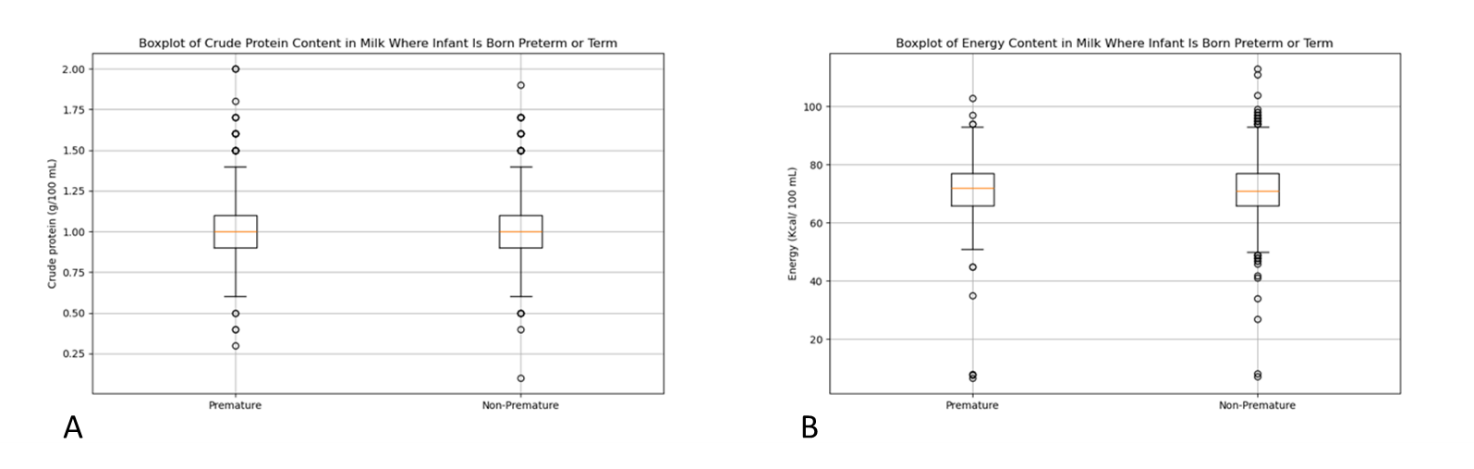


**Figure 3 A-B.** Comparison of protein and energy content from donors whose infants were born preterm or full term. A, boxplots of protein by infants status; B, boxplots of energy by infants status.


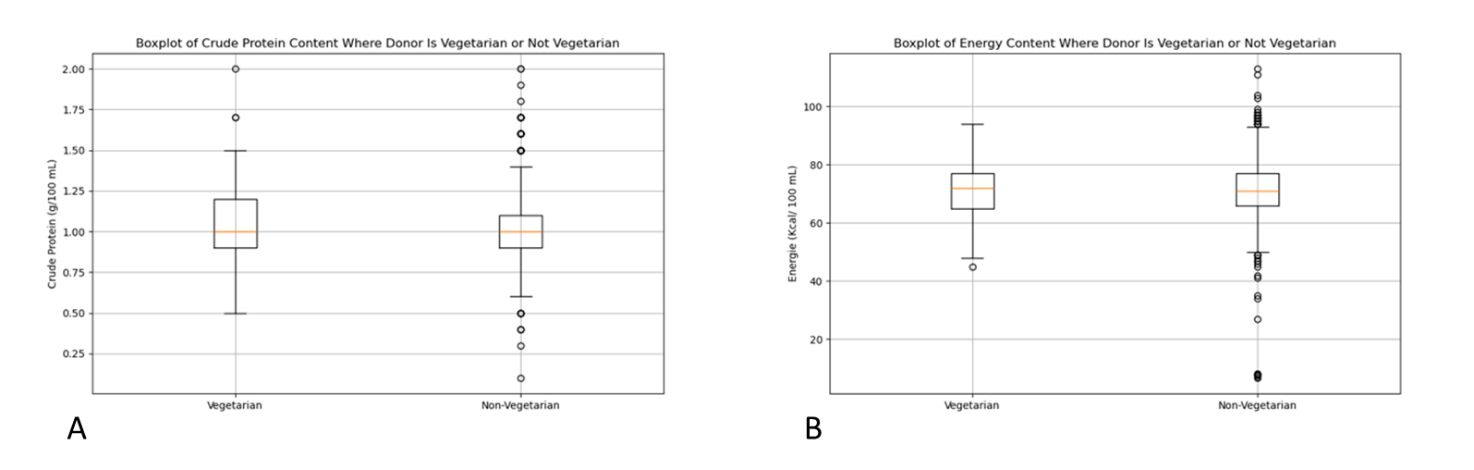


**Figure 4 A-B.** Comparison of protein and energy by donor’s diet. A, boxplots of protein by diet status; B, boxplots of energy by diet status.

**
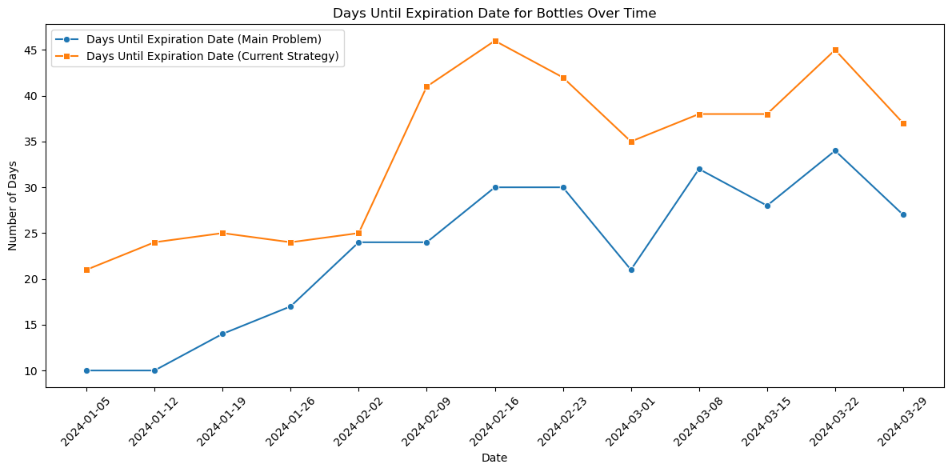
**

**Figure 5.** Comparison of the number of days until bottle expiration, showing the maximum expiration dates for the Main Problem and Current Strategy over time.


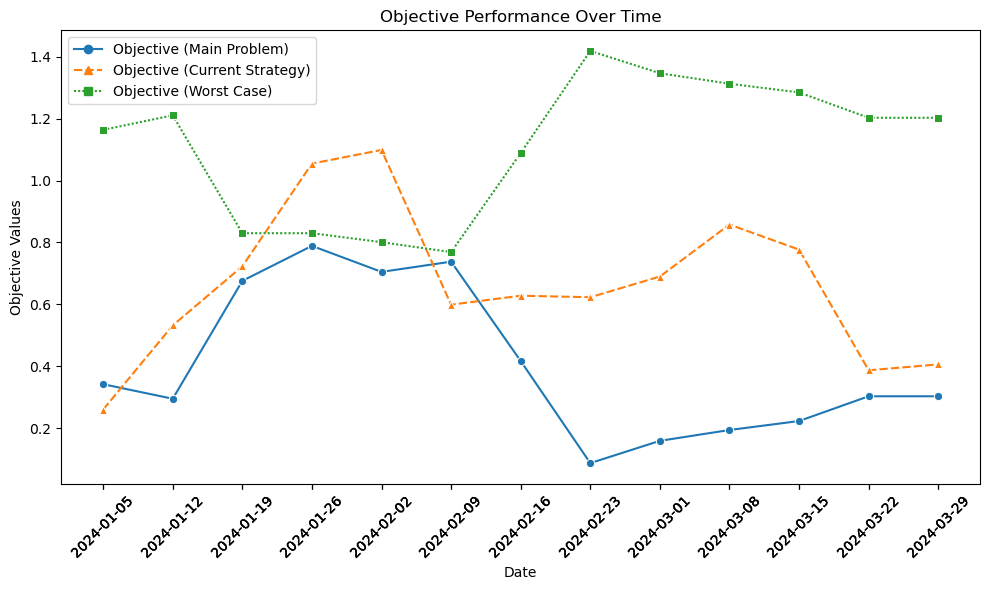


**Figure 6.** Objective performance over time, comparing values for the Main Problem, Current Strategy, and Worst Case across multiple dates.


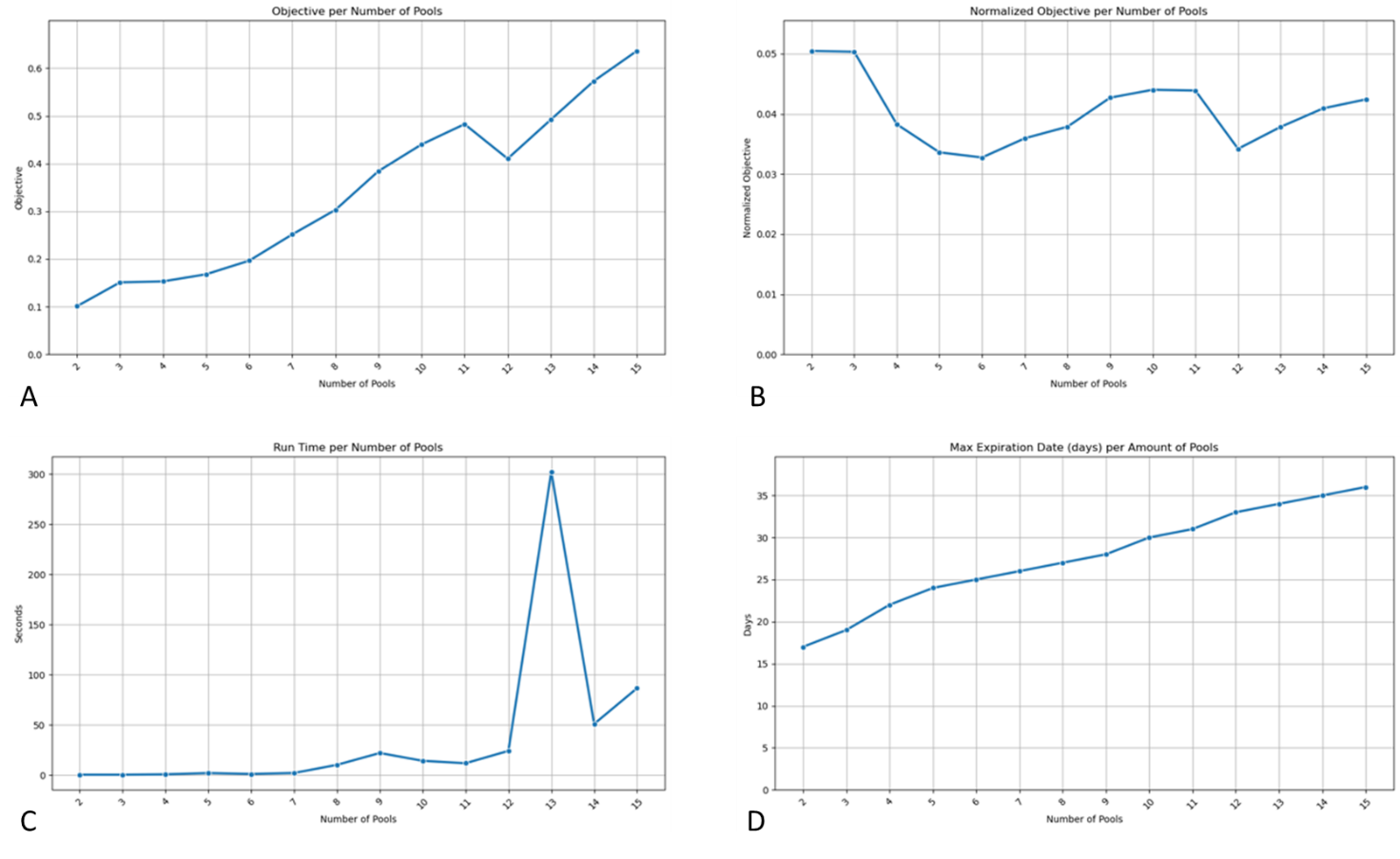


**Figure 7 A-D**. Optimisation results per number of pools. A, objective; B, normalized objective; C, run time; D, maximum expiration date.
