# Supplementary material for: Improving the composition of donor milk using machine learning and optimisation techniques
Source: PLoS One. 2026 Mar 24;21(3):e0345653. doi: 10.1371/journal.pone.0345653 (PMC13012482; doi:10.1371/journal.pone.0345653)
Supplement: S2 File — (DOCX) [file pone.0345653.s002.docx]

**Table 1**. Evaluation Metrics for predicting crude protein content.

|  | **MAE ± SD** | **MAPE** | **RMSE** |
| --- | --- | --- | --- |
| Linear model | 0.180 ± 0.005 | 19.5% | 0.226 |
| Random forest regression | 0.112 ± 0.040 | 11.7% | 0.155 |
| Lasso least angle | 0.152 ± 0.005 | 15.9% | 0.196 |
| Gradient-boosting decision tree | 0.117 ± 0.034 | 12.2% | 0.160 |
| SVM | 0.126 ± 0.020 | 13.1% | 0.168 |
| AdaBoost | 0.130 ± 0.039 | 13.6% | 0.171 |
| Ensemble | 0.114 ± 0.046 | 11.9% | 0.157 |

MAE, mean absolute error; MAPE, mean absolute percentage error; RMSE, root mean square error.

**Table 2**. Evaluation metrics for predicting energy content.

|  | **MAE ± SD** | **MAPE** | **RMSE** |
| --- | --- | --- | --- |
| Linear model | 8.316 ± 0.324 | 12.2% | 10.441 |
| Random forest regression | 5.34 ± 0.038 | 11.2% | 7.231 |
| Lasso least angle | 6.953 ± 0.163 | 13.4% | 9.783 |
| Gradient-boosting decision tree | 5.540 ± 0.039 | 11.4% | 8.991 |
| SVM | 5.751 ± 0.030 | 11.7% | 8.769 |
| AdaBoost | 6.330 ± 0.046 | 12.5% | 9.696 |
| Ensemble | 5.66 ± 0.046 | 11.6% | 8.562 |

MAE, mean absolute error; MAPE, mean absolute percentage error; RMSE, root mean square error.

**Table 3.** Optimisation results for different amount of donors

MIP Gap is the relative difference between the best found solution and the theoretical lower bound, indicating how close the obtained solution is to the optimal solution.

| Number of Donors | Objective Value | Lower Bound | MIP Gap (%) | Runtime (s) | Average Amount Donors | Max Expiration Date (days) |
| --- | --- | --- | --- | --- | --- | --- |
| 1–2 | 0.327 | 0.262 | 19.994 | 302 | 2.000 | 29 |
| 1–3 | 0.305 | 0.301 | 1.152 | 302 | 3.000 | 27 |
| 1–4 | 0.311 | 0.301 | 3.250 | 301 | 4.000 | 27 |
| 1–5 | 0.304 | 0.301 | 0.890 | 17 | 5.000 | 27 |
| 2–3 | 0.302 | 0.301 | 0.202 | 13 | 3.000 | 27 |
| 2–4 | 0.303 | 0.301 | 0.635 | 11 | 3.625 | 27 |
| 2–5 | 0.304 | 0.301 | 0.992 | 4 | 4.875 | 27 |
| 3–4 | 0.304 | 0.301 | 0.860 | 4 | 3.750 | 27 |
| 3–5 | 0.303 | 0.301 | 0.532 | 13 | 5.000 | 27 |
| 4–5 | 0.304 | 0.301 | 0.982 | 4 | 4.625 | 27 |
| 4–6 | 0.303 | 0.301 | 0.608 | 6 | 6.000 | 27 |
