# Supplementary material for: Improving the composition of donor milk using machine learning and optimisation techniques
Source: PLoS One. 2026 Mar 24;21(3):e0345653. doi: 10.1371/journal.pone.0345653 (PMC13012482; doi:10.1371/journal.pone.0345653)
Supplement: S1 Text — (DOCX) [file pone.0345653.s003.docx]

Supplementary material

# Prediction Model

# This study explored both linear and decision tree models to develop a robust prediction model, leveraging the strengths of each approach for enhanced predictive accuracy. Linear models, including linear regression and least angle regression (LARS), were employed for their ability to manage multicollinearity and offer interpretable outputs. Linear regression provides a foundational approach to capturing linear relationships ^1^, while Lasso LARS integrates feature selection and regularization to prevent overfitting, especially in high-dimensional datasets ^2^. Decision tree models like random forest regression and gradient-boosting decision trees were selected for their capability to capture complex, nonlinear interactions and their resistance to overfitting ^3^. Random forest uses an ensemble of decision trees to produce mean predictions, while gradient boosting builds sequential trees to iteratively correct errors, enhancing predictive performance ^4-6^. AdaBoosting focuses on improving misclassified instances, making it effective for imbalanced datasets, and support vector machines (SVMs) were considered for their proficiency with high-dimensional data ^7, 8^. To leverage the strengths of multiple models, an ensemble approach combining random forest and gradient boosting was implemented, enhancing accuracy and robustness through methods like voting regressors ^9^.

# Optimisation Model for the Pooling Process at the HMB

**2.1 Introduction to linear optimisation**

Mathematical Optimisation is often used in practice to solve decision problems for which the number of potential solutions is huge. Just enumerating all potential solutions often leads to unacceptable calculation times. Even using such an enumeration method for seemingly small and simple decision problems may lead to millions years of calculation time. Optimisation could reduce the calculation to minutes or even seconds. An Optimisation approach starts with developing a mathematical model of the decision problem, and then use algorithms to solve it. Such an algorithm avoids complete enumeration, and search in an efficient way for the optimal solution.

In many of the cases a linear optimisation model suffices; this is also the case for the pooling problem. It is called linear since all the mathematical functions contained in the model are linear in the so-called decision variables. The decision variables are the unknowns, and represent the decisions that we want to optimise. Besides the variables, an optimisation model also contains parameters, which are values in the model that are known before we optimise. A linear optimisation model has a linear objective that has to be maximized or minimized, and linear inequalities or equalities. If some of the variables are restricted to be integer or binary, the model is called a mixed-integer linear optimisation model.

**2.2 Variables**

The model for optimising the pooling process at the HMB relies on three key sets. The available $I$ bottles at the moment of pooling are indexed by the set $\mathcal{I=}\{1,2,\ldots,I\}$. The set of $J$ different donors corresponding to the available bottles is $\mathcal{J=}\{1,2,\ldots,J\}$. Lastly, the set of $P$ pools that have to be made is $\mathcal{P=}\{1,2,\ldots,P\}$.

The HMB has several requirements for pooling different bottles of milk together. To model these requirements, we include decision variables to ensure the proper selection of bottles of milk from various donors. The primary decision variable $x_{ip}$ indicates whether bottle $i$ was chosen for pool $p$, where $x_{ip}=1$ means that the bottle was chosen. Another binary variable, $w_{jp}$, indicates whether donor $j$ was used in pool $p$, where $w_{jp}=1$ means that the donor was chosen. For any donor $j$, let $\mathcal{I}_{\mathcal{j}}$denote the subset of deposits that belong to that donor.

**2.3 Objective**

The primary objective of the HMB is to achieve consistent macronutrient quality in pools of human milk. Since the macronutrient content of human milk is only measured after the pooling process is complete, predictions $\text{cp}_{i}$ and $\text{e}_{i}$ were used to estimate the crude protein and energy content, respectively, of a donated bottle $i.$

These predictions were made by two different random forest models: one for crude protein content and one for energy content, with hyperparameters found by grid search. The models were trained on the entire pool dataset to provide accurate estimations. The corresponding volume of a bottle$i$ is denoted by $v_{i}$.

The target values for the macronutrient contents of the resulting pools are $cp_{\text{target}}=$ 1 g/100 mL and $e_{\text{target}}=$ 70 Kcal/100 mL for crude protein and energy content, respectively. Additionally, the target volume for a pool is $v_{\text{tot}}=2$ litres, which is the standard volume of a bottle used in the pooling process at the HMB.

The objective function ensures that the actual contents of the pool are as close as possible to the target values:

$$\min_{x_{ip},w_{jp}} \quad\sum_{p\in\mathcal{P}} \left| 1-\frac{\sum_{i\in\mathcal{I}} x_{ip}\text{cp}_{i}v_{i}}{\text{cp}_{\text{target}}v_{\text{tot}}} \right|+\left| 1-\frac{\sum_{i\in\mathcal{I}} x_{ip}\text{e}_{i}v_{i}}{\text{e}_{\text{target}}v_{\text{tot}}} \right|.$$

It minimises the absolute deviations from the target crude protein and energy contents across all pools. This ensures that the macronutrient content of the pooled human milk remains as close as possible to the desired target values, achieving consistent quality across pools.

This objective function contains absolute values and is therefore nonlinear. However, by introducing extra variables and constraints, we can reformulate this objective to a linear one. The new objective function is

$$\min_{x_{ip},w_{jp},y_{p},z_{p}} \quad\sum_{p\in\mathcal{P}} y_{p}+z_{p},$$

where $y_{p}$and $z_{p}$ are auxiliary variables introduced for the crude protein and energy expressions, respectively. In addition, four constraints were added to the model to ensure that the auxiliary variables correctly represent the absolute deviations:

$$y_{p}\geq1-\frac{\sum_{i\in I} x_{ip}\text{cp}_{i}v_{i}}{\text{cp}_{\text{target}}v_{\text{tot}}},\quad\forall p\in\mathcal{P}$$

$$y_{p}\geq\frac{\sum_{i\in I} x_{{ip\text{cp}}_{i}}v_{i}}{\text{cp}_{\text{target}}v_{\text{tot}}}-1,\quad\forall p\in\mathcal{P}$$

$$z_{p}\geq1-\frac{\sum_{i\in I} x_{{ip\text{e}}_{i}}v_{i}}{\text{e}_{\text{target}}v_{\text{tot}}},\quad\forall p\in\mathcal{P}$$

$$z_{p}\geq\frac{\sum_{i\in I} x_{ip}\text{e}_{i}v_{i}}{\text{e}_{\text{target}}v_{\text{tot}}}-1,\quad\forall p\in\mathcal{P}.$$

**2.4 Constraints**

The optimisation model for the HMB includes several constraints to ensure that the pooling process adheres to the requirements. The HMB uses bottles with a capacity of 2 litres during pooling and therefore, the total volume of a pool must not exceed the capacity. Specifically, this constraint is represented by the following equation:

$$v_{min}\leq\sum_{i\in\mathcal{I}} v_{i}x_{ip}\leq v_{max},\quad\quad\forall p\in\mathcal{P,}$$

where $v_{min}$ represents the minimum allowable volume in one pool, set to 2 litres to guarantee that there is always enough milk in each pool. Conversely, $v_{max}$ represents the maximum allowable volume in one pool and is set to 2.05 litres to account for variations in bottle volume and to provide a margin of flexibility within the model. This constraint ensures that the model consistently follows the correct volume specifications.

We decided that a pool would consist of milk from three to five different donors. Therefore, the model includes constraints to control the number of donors involved in each pool. The first constraint ensures that the number of donors in any pool p falls within the minimum ($n_{min}$) and maximum ($n_{max}$) number of donors allowed per pool, as shown in the following equation:

$$n_{min}\leq\sum_{j\in\mathcal{J}} w_{jp}\leq n_{max},\quad\forall p\in\mathcal{P.}$$

Additionally, the model includes a constraint to ensure that if a donor $j$ is selected for pool $p$, at least one of the bottles from that donor is used in that pool:

$$\sum_{i\in\mathcal{I}_{\mathcal{j}}} x_{ip}\geq w_{jp},\quad\forall j\in\mathcal{J,}p\in\mathcal{P.}$$

Finally, another constraint ensures that a bottle $i$ from donor $j$ can only be included in pool $p$ if donor $j$ is chosen for that pool:

$$x_{ip}\leq w_{jp},\quad\forall i\in\mathcal{I}_{\mathcal{j}},\forall j\in\mathcal{J,}\forall p\in\mathcal{P.}$$

These constraints collectively ensure that the model controls the number of donors per pool and correctly accounts for the inclusion of donors' bottles within each pool.

To prevent any bottle of milk from being used in more than one pool, the model includes the following constraint, which ensures that each bottle $i$ is assigned to at most one pool:

$$\sum_{p\in\mathcal{P}} x_{ip}\leq1,\quad\forall i\in\mathcal{I.}$$

Finally, the decision variables constraints, which establish that $x_{ip}$and $w_{jp}$ are binary and $y_{p}$ and $z_{p}$ are non-negative, are as follows:

$$x_{ip},w_{jp}\in\{0,1\},\quad y_{p},z_{p}\geq0,\quad\forall i\in\mathcal{I,}\forall j\in\mathcal{J,}\forall p\in\mathcal{P.}$$

To minimise waste at the HMB, all bottles with more days remaining before expiration than a specified threshold $\text{D}^{*}$ were excluded from all pools, as shown in the following constraint:

$$x_{ip}=0,\quad\forall i\in\mathcal{I,}\forall p\in\mathcal{P |}d_{i}>D^{*}.$$

In this context, parameter $d_{i}$ represents the remaining days until the expiration date of bottle $i$ , and $\text{D}^{*}$ is the threshold for the maximum number of remaining days for a bottle to be included in any pool. This constraint ensures that any bottle with more remaining days than the threshold is not included in any pool, thereby prioritizing bottles that are close to their expiration date.

The optimal value for $\text{D}^{*}$ changes each time the HMB starts their pooling process, and it is therefore crucial that this value be recalculated each time. This is done by running a preliminary optimisation model, where the first step minimises the total remaining days until expiration for all bottles in all pools, formulated as follows:

$$\min_{x_{ip},w_{jp}} \sum_{p\in\mathcal{P}} \sum_{i\in\mathcal{I}} d_{i}x_{ip}.$$

This creates a two-step optimisation model. The first step solves the above equation using constraints from the volume constraint to the decision variable constraint. After this model is solved, the largest value for $d_{i}$ that is used in one of the pools of the solution is used as the value for $\text{D}^{*}$ in the second stage of the optimisation model, and the expiration constraint is added to the model.

**2.5 Full model**

The final mixed-integer linear optimisation model is the following:

$$\min_{x_{ip},w_{jp},y_{p},z_{p}} \quad\sum_{p\in\mathcal{P}} y_{p}+z_{p}$$

$${such that y}_{p}\geq1-\frac{\sum_{i\in I} x_{ip}\text{cp}_{i}v_{i}}{\text{cp}_{\text{target}}v_{\text{tot}}},\quad\forall p\in\mathcal{P}$$

$$y_{p}\geq\frac{\sum_{i\in I} x_{{ip\text{cp}}_{i}}v_{i}}{\text{cp}_{\text{target}}v_{\text{tot}}}-1,\quad\forall p\in\mathcal{P}$$

$$z_{p}\geq1-\frac{\sum_{i\in I} x_{{ip\text{e}}_{i}}v_{i}}{\text{e}_{\text{target}}v_{\text{tot}}},\quad\forall p\in\mathcal{P}$$

$$z_{p}\geq\frac{\sum_{i\in I} x_{ip}\text{e}_{i}v_{i}}{\text{e}_{\text{target}}v_{\text{tot}}}-1,\quad\forall p\in\mathcal{P}$$

$$v_{min}\leq\sum_{i\in\mathcal{I}} v_{i}x_{ip}\leq v_{max},\quad\quad\forall p\in\mathcal{P}$$

$$n_{min}\leq\sum_{j\in\mathcal{J}} w_{jp}\leq n_{max},\quad\forall p\in\mathcal{P}$$

$$\sum_{i\in\mathcal{I}_{\mathcal{j}}} x_{ip}\geq w_{jp},\quad\forall j\in\mathcal{J,}p\in\mathcal{P}$$

$$x_{ip}\leq w_{jp},\quad\forall i\in\mathcal{I}_{\mathcal{j}},\forall j\in\mathcal{J,}\forall p\in\mathcal{P}$$

$$\sum_{p\in\mathcal{P}} x_{ip}\leq1,\quad\forall i\in\mathcal{I}$$

$$x_{ip}=0,\quad\forall i\in\mathcal{I,}\forall p\in\mathcal{P |}d_{i}>D^{*}$$

$$x_{ip},w_{jp}\in\{0,1\},\quad y_{p},z_{p}\geq0,\quad\forall i\in\mathcal{I,}\forall j\in\mathcal{J,}\forall p\in\mathcal{P.}$$

# Solver and Implementation Details

To implement the optimisation model, we utilised the Pyomo library, a Python-based open-source optimisation modelling language. Pyomo provides a flexible and expressive way to define and solve optimisation problems. To find the optimal solution, we used the Gurobi solver, which is well-known for its efficiency and capability to handle large mixed-integer linear optimization problems. The gap tolerance for the solver was set to 0.01, which means the solver will stop when it finds a solution with objective value within 1% of the optimal objective value. Additionally, we imposed a time limit of 300 seconds for the solver. The optimality gap reported by the solver appeared to be 0.65%. The runtime for the solver averaged 44 seconds, with most cases having a shorter runtime and only three instances taking longer. The runtime was calculated by measuring the time taken to solve the first two steps of the optimisation model, in which the maximum expiration date (D∗) was determined and the pools were established. The optimisation model was implemented and solved on a MacBook Air with an M3 chip and 16 GB of RAM.

**References**

1. Montgomery DC, Peck, E. A., & Vining, G. G.,. Introduction to linear regression analysis (6th ed): Wiley-interscience; 2021.

2. Bradley E, Trevor H, Iain J, Robert T. Least angle regression. The Annals of Statistics. 2004;32(2):407-99.

3. Breiman L. Random Forests. Machine Learning. 2001;45(1):5-32.

4. Biau G, Scornet E. A random forest guided tour. TEST. 2016;25(2):197-227.

5. Jerome HF. Greedy function approximation: A gradient boosting machine. The Annals of Statistics. 2001;29(5):1189-232.

6. Natekin A, Knoll A. Gradient boosting machines, a tutorial. Front Neurorobot. 2013;7:21.

7. Freund Y, Schapire RE. A Decision-Theoretic Generalization of On-Line Learning and an Application to Boosting. Journal of Computer and System Sciences. 1997;55(1):119-39.

8. Cortes C, Vapnik V. Support-vector networks. Machine Learning. 1995;20(3):273-97.

9. Kyriakides G, & Margaritis, K. G.,. Hands-on ensemble learning with Python: Packt Publishing Ltd; 2019.
